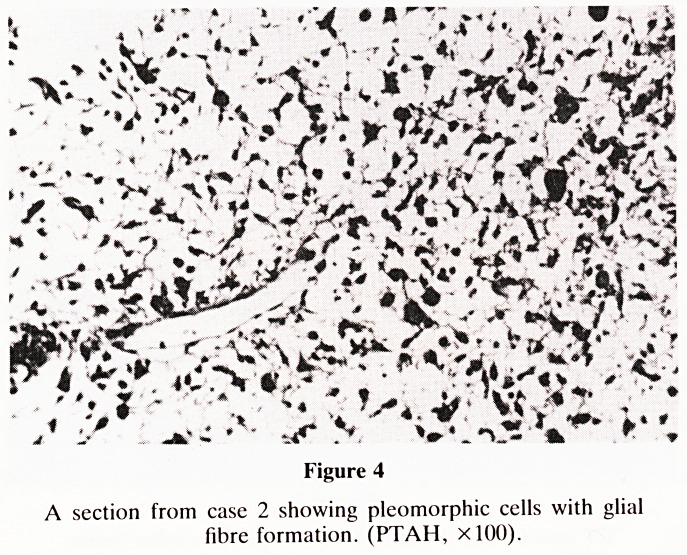# Cerebellar Glioblastoma Multiforme; a Report of Two Cases and Review of the Literature

**Published:** 1990-06

**Authors:** Tipu Zahed Aziz, Mark Stoddart

**Affiliations:** Department of Neurosurgery, University Hospital of Wales, Cardiff; Department of Neurosurgery, University Hospital of Wales, Cardiff

## Abstract

Cerebellar Glioblastoma Multiforme is a rare condition, a review of the world literature and two further cases are presented.Cerebellum, gliablastoma multiforme, surgery, radiotherapy.


### Cerebellar Glioblastoma Multiforme; A Report of Two Cases and Review of the Literature

**Tipu Zahed Aziz, BSc, MB BS, FRCS**

**Mark Stoddart BSc, MBBS**
Department of Neurosurgery, University Hospital of Wales, Cardiff

**SUMMARY**
Cerebellar Glioblastoma Multiforme is a rare condition, a review of the world literature and two further cases are presented.Cerebellum, gliablastoma multiforme, surgery, radiotherapy.

### INTRODUCTION

Cerebellar Glioblastoma Multiforme is rare, only 62 well documented cases have been found in the world literature. We report two cases treated in 1988 at the University Hospital of Wales, Cardiff (U.H.W.).

### Case 1

S.W., a 69 year old male was admitted to the Neurosurgical Unit (U.H.W.) on 22.3.88 with a two year history of imbalance, occassional attacks of vertigo associated with nausea. In the two months prior to admission he began to complain of headaches and weakness of both legs but remained ambulant. On examination he had marked truncal ataxia, right sided dysdiadochokinesia, dysarthria and papilloedema. A CT scan (Fig. 1) showed an enhancing mass in the right cerebellar hemisphere with moderate lateral ventricular dilatation.

On the 29.3.88 at posterior fossa craniectomy an ill defined tumour was debulked. He made a good post operative recovery and underwent radiotherapy receiving 4000 Rads to the whole brain followed by 2000 Rads to the tumour bed. He died on 12.2.90, a CT scan prior to demise confirmed recurrent tumour. No autopsy was performed.

### Case 2

A 55 year old male was admitted to the same unit on 30.8.88 with a three month history of unsteadiness of gait, falling to the left, occipital headaches and nausea. On examination he had horizontal nystagmus, left sided dysdiadochokinesia, an ataxic gait veering to the left but no papilloedema. A CT scan (Fig. 2) showed an enhancing mass in the left cerebellar hemisphere with lateral ventricular dilatation.

At posterior fossa exploration on 1.9.88 a soft greyish tumour was decompressed. He made an uneventful recovery and received 4500 Rads whole brain irradiation.

On 4.11.88 his general condition had deteriorated enough to merit re-admission to hospital and he died on 22.11.88. An autopsy was not performed.

### PATHOLOGY

At the time of operation smears were made from both cases and stained with haematoxylin and eosin (H&E). Paraffin sections were also prepared and stained with H&E and PTAH (phosphotungstic acid haematoxylin).

The smear preparation from case 1 revealed pleomorphic poorly differentiated astrocytic cells and scattered giant cells; that from case 2, poorly differentiated malignant cells and occasional multinucleate cells. In each case a preliminary diagnosis of malignant astrocytoma (grade 3–4) was made.

**Figure 1**
CT scan of case 1 which shows the right cerebellar mass.

**Figure 2**
CT scan of case 2 which shows the left cerebellar lesion.

**Figure 3**

A section of the tumour from case 1 showing endothelial proliferation, pleomorphic cells and scattered multinuclate cells (H&E, ×100).

The paraffin sections of case 1 (Fig. 3) revealed a pleomorphic tumour with scattered multinucleate cells. There was marked endothelial cell proliferation and PTAH stains for glial fibres were strongly positive. Sections from case 2 (Fig. 4) revealed fragments of cerebellar tissue infiltrated by poorly differentiated pleomorphic tumour cells and numerous multinucleate cells. Endothelial proliferation was present but not as marked as in case 1. PTAH stains were markedly positive for glial fibres. In both cases a diagnosis of malignant astrocytoma (glioblastoma multiforme) of the cerebellum was made.

### DISCUSSION

Cerebellar Glioblastoma Multiforme is rare, only 64 cases, including the present two cases, have been reported in the world literature (Table 1, refs. 1–21, 23–26, 28–37). The tumour is largely one of adult life but 23 cases have been reported below the age of 20 years (refs. 3, 6, 7, 12, 15, 16, 19, 21, 30). The male/female ratio is nearly 1:1, the average age of onset is 35.5 in females and 35.7 years in males. In patients under 20 years of age, the average age of onset is 11.9 years in males and 9 years in females with a male/female ratio of 1:1.

The survival rates, in weeks, for patients who have undergone surgery alone, including biopsy, partial and complete resection (SR), surgery followed by cranial irradiation (SR + RT) and surgery followed by irradiation and chemotherapy (SR + RT + CT) are shown in Table 2.

**Figure 4**

A section from case 2 showing pleomorphic cells with glial fibre formation. (PTAH, ×100).

**Table 1**

| *Author* | *Year* | *Age* | *Sex* | *Site* | *Rx.* | *Surv.* |
|---|---|---|---|---|---|---|
| Powell | 1947 | 70 | M | LH | Nil | ? |
| Davis | 1949 | 8 | M | RH | Nil | ? |
| | | 12 | M | RH | Nil | ? |
| Ringertz | 1951 | 32 | F | LH | ? | 164 |
| Roth | 1960 | 42 | M | LH | Nil | 7 |
| | | 51 | M | RH | Nil | 33 |
| Huntington | 1965 | 32 | M | LH | Nil | 3 |
| Masucci | 1966 | 30 | M | L,RH.V | BIOP. | 6 |
| Campanella | 1967 | 57 | M | LH.P.V. | BIOP. | 5 |
| Gross | 1968 | 49 | F | V | BIOP. | 18 |
| | | 64 | M | LH | SR | 2 |
| | | 39 | M | LH | CR + RT | 52 |
| Tateishi | 1969 | 65 | F | LH | Nil | 15 |
| Wieczorek | 1971 | 60 | F | LH | Nil | 13 |
| Dohrmann | 1975 | 55 | F | RH | PR + RT | 40 |
| Miller | 1976 | 67 | M | RH | BIOP. | 7 |
| Fresh | 1976 | 9 | F | LH | CR + RT + CT | 130 |
| Kleinman | 1978 | 53 | F | RH | PR | 5 |
| Tamura | 1979 | 33 | F | V | PR + RT | 10 |
| Tibbs | 1980 | 49 | F | RH | PR + RT + CT | 15 |
| Luccarelli | 1980 | 34 | F | RH | PR + RT | 130 + |
| | | 52 | M | LH.P | PR | ? |
| | | 6 | F | LH.IVth | PR | 1 |
| Aun | 1981 | 58 | F | V | BIOP. | ? |
| Auff | 1981 | 54 | F | LH.V.P | Nil | 15 + |
| Escalona | 1981 | 51 | M | L,RH.V | BIOP. | 2 |
| Salazar | 1981 | 8 | F | Not spec. | SR + RT | 55 |
| | | 6 | F | — | — | 60 + |
| | | 7 | M | — | — | 50 |
| | | 10 | M | — | — | 100 |
| | | 9 | M | — | — | 55 |
| | | 16 | M | — | — | 180 + |
| | | 13 | N | — | — | 140 + |
| | | 10 | M | — | — | 180 + |
| | | 9 | M | — | — | 90 |
| | | 11 | M | — | — | 40 |
| | | 9 | F | — | — | 210 + |
| | | 10 | F | — | — | 240 + |
| | | 10 | F | — | — | 100 + |
| Kopelson | 1982 | 15 | M | — | PR + RT | 470 |
| | | 53 | M | — | CR + RT | 25 |
| | | 57 | M | — | PR + RT | 126 |
| | | 60 | M | — | BIOP. + RT | 52 |
| | | 10 | F | — | CR | 4 |
| | | 30 | M | — | PR | 4 |
| | | 54 | F | — | BIOP. | 4 |
| | 67 | M | — | BIOP. | 4 | |
| Zito | 1983 | 77 | F | V | PR + RT | 52 + |
| | | 57 | M | LH | PR + RT | 104 |
| | 76 | F | RH | PR + RT | 10 | |
| | 85 | M | RH | PR | 0.5 | |
| Georges | 1983 | 17 | M | RH | PR + RT 40 | |
| Bhimani | 1983 | 21 | M | RH.V | PR + RT + CT | ? |
| Hegedus | 1983 | 39 | F | RH.V | PR + RT + CT | 290 |
| Chin | 1984 | 14 | F | V | CR + RT | 156 |
| Pombo | 1985 | 9 | F | RH | BIOP. | 1 |
| Yamada | 1986 | 55 | M | RH | SR + RT + CT | 190 |
| | | 27 | F | RH | SR + RT + CT | 125 |
| Inbasekaran | 1986 | 8 | F | V | PR | 1 |
| Levine | 1987 | 80 | F | V | CT + RT | 210 |
| Schmidbauer | 1987 | 21 | M | RH | CR + RT + CT | 104 |

*Abbreviations:*

SURV. survival in weeks from presentation to death.
Rx. treatment.
CR complete resection.
PR partial resection.
SR surgical resection, no details given.
BIOP. biopsy only.
RT radiotherapy.
CT chemotherapy.
LR, RH left and right hemispheres.
V vermis.
P, IV pontine, forth ventricle.

**Table 2**

| | *No. of cases* | *Average survival (weeks)* |
|---|---|---|
| SR | 15 | 4.3 |
| SR + RT | 30 | 100.6 |
| SR + RT + CT | 6 | 142.3 |

**Table 3**

| *Age* | *SR* | *SR + RT* | *SR + RT + CT* |
|---|---|---|---|
| 0–20 years | 1.75 (n = 4) | 129.8 (n = 17) | 130.0 (n = 1) |
| 20–40 years | 18.0 (n = 2) | 49.0 (n = 4) | 173.0 (n = 3) |
| 40–60 years | 8.3 (n = 3) | 52 (n = 7) | 102.5 (n = 2) |
| 60 = years | 3.16 (n = 3) | 63.2 (n = 5) | Nil |

The two cases presented were treated by sub-total resection followed by radiotherapy. The radiotherapy protocols were different in each case. J.T. survived 13 weeks and S.W. 115 weeks after surgery.

Surgery alone can do little to improve survival but when combined with radiotherapy the results are encouraging, particularly since radiotherapy has been shown to increase the survival time and the tumour free interval in supratentorial glioblastoma multiforme (ref. 27). Radiotherapy schedules are often dictated by personal and local preferences. We support the views of Kopelson (refs. 18, 19) who recommends posterior fossa irradiation only. The biological behaviour of these tumours would seem similar to that of the brain stem glioma in that they are locally invasive (ref. 22), and therefore, the risk of exfoliation and distant neuraxial spread, so characteristic of ependymomas and medulloblastomas, is very low. The regimes of total cranio-spinal irradiation in such cases would appear to be excessive.

The role of chemotherapy is less clear. It would appear from the survival figures (table 1) that this may have a useful effect. The studies of the EORTC brain tumour group (refs. 9, 10) have shown that CCNU may extend survival in patients with supratentorial glioblastoma multiforme in conjunction with surgery and radiotherapy.

For all treatment protocols, patients over the age of 60 years generally do less well than younger ones when the average figures, in weeks, of different age groups are examined (table 3).

Ten cases had no treatment and in three cases no survival figures were quoted. A statistical comparison of the various modes of therapy could not be performed as the regimes varied so widely.

### CONCLUSION

It is not possible to make any convincing conclusions about optimal treatment for this tragic condition nevertheless, for several reasons this remains a fascinating histological entity.

Cerebellar astrocytoma is largely a disease of children. If the glioblastoma were a malignant transformation in the childhood astrocytoma the age of presentation should be less than that seen. Very few showed any such predisposing conditions. In two cases the condition developed after posterior fossa irradiation for a benign astrocytoma and medulloblastoma respectively (refs. 17, 31), and in four cases (ref. 30) the glioblastoma occurred in the presence of another tumour, three had concurrent ependymomas and one had an ependymoma and an oligodendroglioma.

The rarity of the condition in the cerebellum far exceeds what one would expect if the smaller size of the cerebellum compared to the cerebral hemispheres were the major factor. The cerebellum is approximately one-tenth of the mass of the cerebral hemispheres.

It may well be that glioblastoma multiforme of the cerebellum is a distinct condition from the supratentorial form. Although histological sections suggest them to be identical, the tissue culture studies of Escalona-Zapata (ref. 9) suggest that it may arise from cerebellar astrocytes in a selective fashion. It may explain the inconsistencies of this curious but difficult neurosurgical enigma.

### ACKNOWLEDGEMENTS

We are grateful to Mr. R. D. Weeks at the U.H.W. for permission to report these two cases and to Dr. G. Coles and the Department of Pathology at the U.H.W. for the neuropathological studies.

### REFERENCES

1. AUFF E. and VASS K. (1981) Cerebellar glioblastome presenting clinically as Wallenburgs syndrome. *Arch. Psychiatr. Nervenkr.* **230**, 361–364.
2. AUN R. A., STAVALE J. M. and SILVA D. (1981) Glioblastoma Multiforme no cerebelo. *Arq. Neuropsiquiatr.*, Sept., **39**(3), 350–354.
3. BABSON FRESH C., TAKEI Y. and O'BRIEN M. S. (1976) Cerbellar glioblastoma in childhood. *J. Neurosurg. Dec.* **45**, 705–708.
4. BHIMANI S., VIRAPONGSE C., SPENCER D. and KIM J. (1983) CT appearance of cerebellar glioblastoma multiforme. *J. of Comp. Asst. Tomog.* Oct **7**(5), 889–891.
5. CAMPANELLA G. (1967) Una rara localizzazione del glioblastoma multiforme nell'adulto. *Acta Neurol. (Napoli)* **22**(1), 87–95.
6. CHIN H. W., MARUYAMA Y. and TIBBS P. (1984) Cerebellar glioblastoma in childhood. *J. Neuro oncol.* 79–84.
7. DAVIS L., MARTIN J. and GOLDSTEIN S. L. (1949) A study of 211 patients with veried glioblastoma multiforme. *J. Neurosurg.* **6**, 33–44.
8. DOHRMANN G. J. and DUNSMORE R. H. (1975) Glioblastoma multiforme of the cerebellum. *Surg. Neurol. Apr.* **3**, 219–223.
9. ESCALONA-ZAPATA J., SALINERO E. and LACRUZ C. (1981) Malignant cerebellar gliomas. *J. Neurosurg. Sci.* **25**, 95–103.
10. EORTC brain tumour group (1981) Evaluation of CCNU, VM–26 + CCNU + Procarbazine in supratentorial brain gliomas. *J. Neurosurg.* **55**, 27–31.
11. EORTC brain tumour group (1978) Effect of CCNU on survival rate of objective remission and duration of free interval in patients with malignant brain glioma. *Europ. J. Cancer* **14**, 851–856.
12. GEORGES P. M., NOTERMAN J. and FLAMENT-DURAND J. (1983) Glioblastoma of the cerebellum in children and adolescents. *J. Neuro oncol.* **1**, 275–278.
13. GROSS S., COHEN R. and PANICHAVANTANA S. (1969) Cerebellar glioblastomas. *J. Mt. Sinai Hosp. New York* **32**(2), 123–129.
14. HEGEDUS K. and MMLNAR P. (1983) Primary cerebellar glioblastoma multiforme with an unusually long survival. *J. Neurosurg.* **58**, 589–592.
15. HUNTINGTON R. W., CUMMINGS K.L. and MOE T. I. (1965) Discovery of fatal primary intracranial neoplasms at medico-legal autopsies. *Cancer* **18**, 117–127.
16. INBASEKARAN V., JAWAHAR G. and NATARAJAN M., (1986) Cerebellar glioblastoma. *J. Indian M.A.* **84**, 279–280.
17. KLEINMAN G. M., SCHOENE W. C. and WALSHE T. M. (1978): Malignant transformation in benign cerebellar astrocytoma. *J. Neurosurg.* **49**, 111–118.
18. KOPELSON G. (1982) Cerebellar glioblastoma. *Cancer* **50**, 308–311.
19. KOPELSON G. and LINGGOOD R. (1982) Infratentorial glioblastoma: the role of neuraxis irradiation. *Int. J. Oncology Biol. Phys.* **8**, 999–1003.
20. LEVINE G. A., McKEEVER P. E. and GREENBERG H. S. (1987) Primary cerebellar glioblastoma multiforme. *J. Neuro. oncol.* **5**, 231–236.
21. LUCCARELLI G. (1980) Glioblastoma of the cerebellum: description of three cases. *Acta Neurochir.* **53**, 107–116.

22. R.V.P., PHATAK R. and BELLUR S. (1982) Brain stem gliomas, an autopsy study of 25 cases. *Cancer,* **49**, 1294–1296.
23. MASUCCI E. F., FERRERO A. A. and KURTZE J. F. (1966) Glioblastoma multiforme invadind the posterior fossa. *Dis. Nerv. Sys.* **27**, 47–51.
24. MILLER E. M., MANI R. L., TOWNSEND J. J. (1976) Cerebellar glioblastoma in an adult. *Surg. Neurol.* **5**, 341–343.
25. POMBO R., TORTELLY-COSTA A. and BULACIO E. (1985) Glioblastoma multiforme cerebellar. *Arq. Neuropsiquiatr.* **43**(1), 102–107.
26. POWELL C. B. (1947) Primary glioblastoma multiforme of the cerebellum. A case report. *J. Neuropath. Exp. Neurol.* **6**, 279–285.
27. RAMSAY R. G. and BRAND W. M. (1973) Radiotherapy of glioblastoma multiforme. *J. Neurosurg.* **39**, 197–202.
28. RINGERTZ N. and NORDENSTAM H. (1951) Cerebellar astrocytoma. *J. Neuropath. Exp. Neurol.* **10**(4), 343–367.
29. ROTH J. G. and ELVIDGE A. R. (1960) Glioblastoma multiforme, a clinical survey. *J. Neurosurg.* **17**, pp.736–750.
30. SALAZAR O. M. (1981) Primary malignant cerebellar astrocytomas in children, a signal for post operative craniospinal irradiation. *Int. J. Oncology Biol. Phys.* **7**, 1661–1665.
31. SCHMIDBAUER M., BUDKA H. and BRUCKNER R. (1987) Glioblastoma developing at the site of a cerebellar medulloblatoma treated 6 years earlier. *J. Neurosurg.* **67**, 915–918.
32. TAMURA M., KAWAKUCHI J. and WAKAO T. (1979) Cerebellar glioblastoma, a case report. *Neurol. Med. Chir. (Tokyo)* **19**, 517–522.
33. TATEISHI J., KONO M. and MURAKAMI M. (1970) Clinico-pathological study of an adult case of cerebellar glioma. *No-To-Shinkei.* **22**(2), 183–189.
34. TIBBS P. A., MORTARA R. H. (1980) Primary glioblastoma multiforme of the cerebellum, a case report. *Acta Neurochir.* **52**, 13–18.
35. WIECZOREK V., HEID R. and BOCK R. (1971) Ungewohnliche Liquorzellveranderungen bei einem Kleinhirntumor. *Nervenarzt.* **42**, 270–273.
36. YAMADA S., AIBA T. and HARA M. (1986) Primary glioblastoma of the cerebellum, a case report. *Neurol. Med. Chir. (Tokyo)* **26**, 233–239.
37. ZITO J., SIVA A., SMITH T. W. (1983) Glioblastoma of the cerebellum. *Surg. Neurol.* **19**, 373–378.

## Figures and Tables

**Figure 1 f1:**
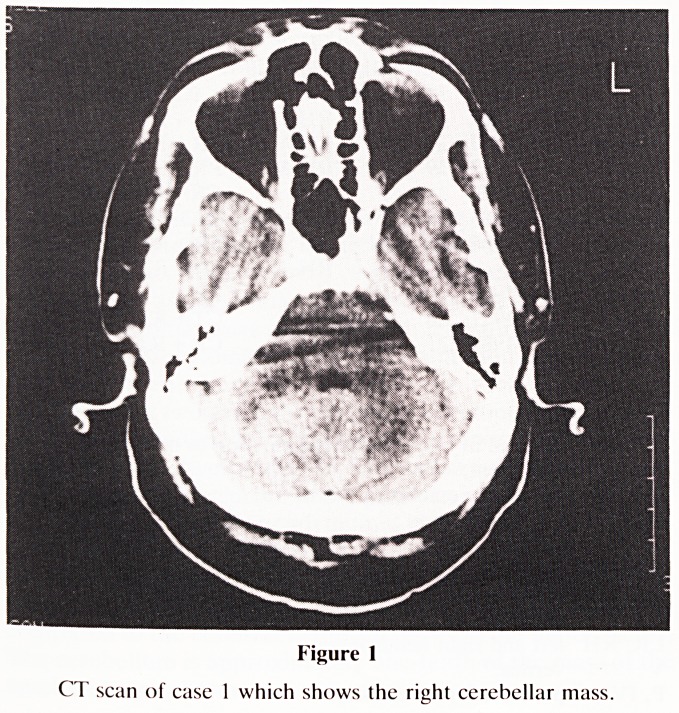


**Figure 2 f2:**
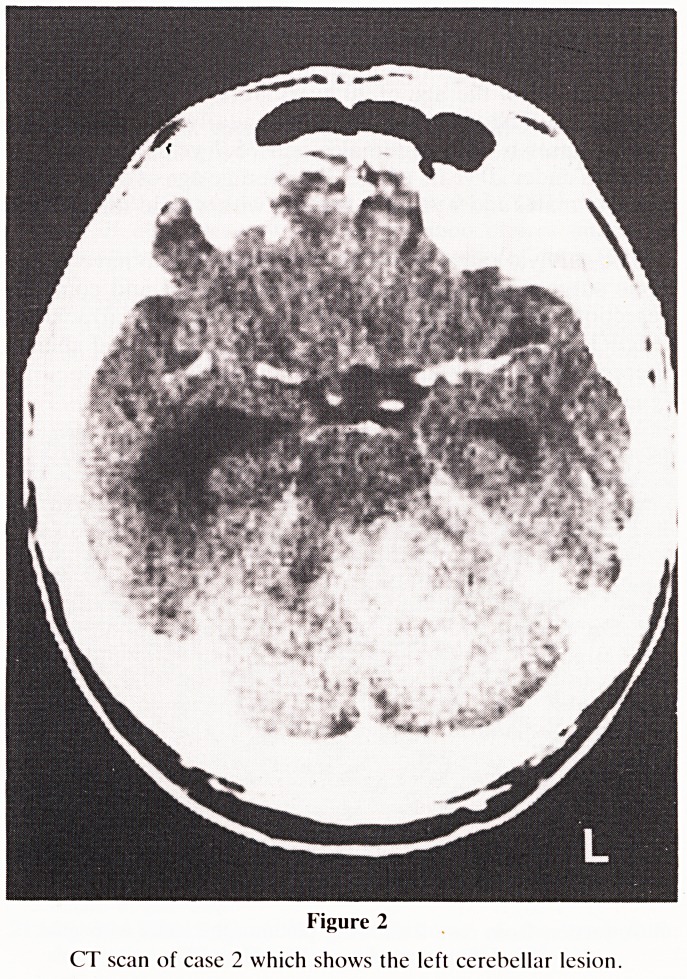


**Figure 3 f3:**
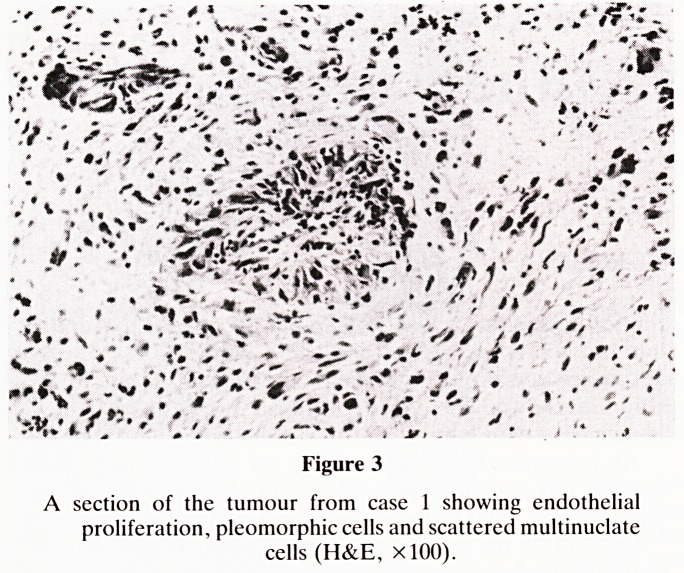


**Figure 4 f4:**